# Assessing Brain–Muscle Connectivity in Human Locomotion through Mobile Brain/Body Imaging: Opportunities, Pitfalls, and Future Directions

**DOI:** 10.3389/fpubh.2018.00039

**Published:** 2018-02-26

**Authors:** Federico Gennaro, Eling D. de Bruin

**Affiliations:** ^1^Institute of Human Movement Sciences and Sport, Department of Health Sciences and Technology, ETH Zurich, Zurich, Switzerland; ^2^Division of Physiotherapy, Department of Neurobiology, Care Sciences and Society, Karolinska Institutet, Stockholm, Sweden

**Keywords:** mobile brain/body imaging, independent component analysis, corticomuscular coherence, neural drive, gait, electroencephalography, central pattern generators, age-related sarcopenia

## Abstract

Assessment of the cortical role during bipedalism has been a methodological challenge. While surface electroencephalography (EEG) is capable of non-invasively measuring cortical activity during human locomotion, it is associated with movement artifacts obscuring cerebral sources of activity. Recently, statistical methods based on blind source separation revealed potential for resolving this issue, by segregating non-cerebral/artifactual from cerebral sources of activity. This step marked a new opportunity for the investigation of the brains’ role while moving and was tagged mobile brain/body imaging (MoBI). This methodology involves simultaneous mobile recording of brain activity with several other body behavioral variables (e.g., muscle activity and kinematics), through wireless recording wearable devices/sensors. Notably, several MoBI studies using EEG–EMG approaches recently showed that the brain is functionally connected to the muscles and active throughout the whole gait cycle and, thus, rejecting the long-lasting idea of a solely spinal-driven bipedalism. However, MoBI and brain/muscle connectivity assessments during human locomotion are still in their fledgling state of investigation. Mobile brain/body imaging approaches hint toward promising opportunities; however, there are some remaining pitfalls that need to be resolved before considering their routine clinical use. This article discusses several of these pitfalls and proposes research to address them. Examples relate to the validity, reliability, and reproducibility of this method in ecologically valid scenarios and in different populations. Furthermore, whether brain/muscle connectivity within the MoBI framework represents a potential biomarker in neuromuscular syndromes where gait disturbances are evident (e.g., age-related sarcopenia) remains to be determined.

## Central Pattern Generators (CPGs) or Cortical Mechanisms? A Long-Lasting (Open) Question in Motor Control of Gait

**Figure 1:**
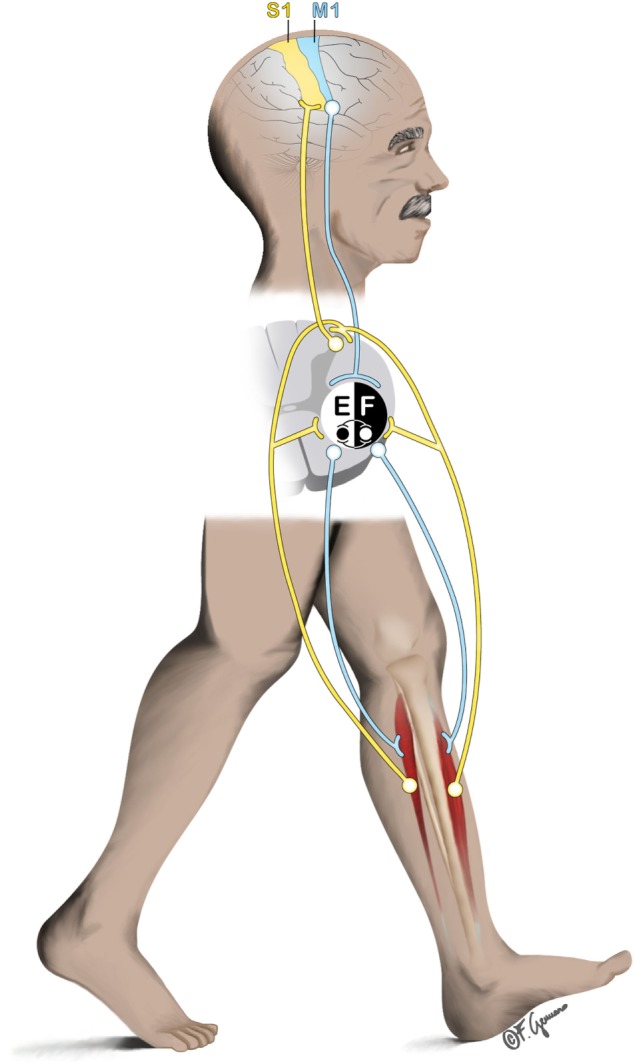
General representation of the interplay between supraspinal (primary sensorimotor cortex), spinal (central pattern generators), and subspinal structures (e.g., ankle dorsiflexors) during locomotion. Efferent pathway from the primary motor cortex (M1) descends to the spinal cord to initiate/mediate the gait. At spinal cord level, contralateral ankle flexors and extensors (here represented by the tibialis anterior [F]; and Soleus [E] respectively) are excited/inhibited rhythmically to produce the basic locomotor pattern of gait. Afferent pathway (e.g., originating from the muscles) ascends to the spinal cord and to the contralateral primary sensory cortex (S1) to produce feedback corrections of the locomotor patterns.

Whether human locomotion is driven primarily by supraspinal or subcortical mechanisms within the central nervous system (CNS) is object of long-lasting debates. In mammalians, muscle activation requires CNS inputs either dependently (1) or independently (2) from peripheral sensory feedback based on whether locomotion is actually performed or not. This finding is based on animal experimental data from the beginning of the nineteenth (3, 4) and twentieth centuries (5). These empirical findings forged the core of the CPGs, spinal interneuronal networks, theory (6). Since then and until recently, CPGs were considered playing the most crucial role in human locomotion. Briefly, CPGs model human locomotion (i.e., walking) as an action initiated by the brain, but yet are maintained (and constrained) in its steady-state execution by mostly spinal mechanisms with the interaction of peripheral afferent contributions (1). However, intentional gait modifications require motor programming in the premotor cortices (7) and, thus, indicate a role for supraspinal control of human walking. Here, we summarize recent studies on human walking showing that bipedalism is achieved by inputs and modifications from supraspinal, spinal, and peripheral structures (Figure 1). The corresponding contributory weights of the different structures that help in controlling different modes of locomotion (e.g., running, walking with or without cognitive dual-tasking), remain to be explored. Recent advances in technology lead to the hypothesis that the control of human bipedalism can be explored by applying experimental approaches that allow imaging of human brain dynamics in actively moving individuals.

A constant motor control of human bipedalism has already been hypothesized two centuries ago by scientists such as George Hayward, who wrote: “*The nerves convey the stimulus of volition to the muscles* … *and the whole machine is thus put in motion under the guidance of the will*” (8). Nevertheless, at that time such a theory could not be demonstrated experimentally, given that a device able to record brain activity was not yet available.

## Assessing the Brain During Movement: Opportunities and Pitfalls

Only one century later Hans Berger recorded the first electroencephalography (EEG) from the human scalp (9). Since then and almost until the end of the 20th century, EEG has been involved in several studies on physiological and pathological neural mechanisms, however, it was not yet usable for assessing the supraspinal role in human locomotion. Each EEG electrode measures electrical potentials from a huge neuronal pool (e.g., from thousands of neurons) and this neuronal activity is spread out because of the volume conduction within the skull. Therefore, at the end each EEG electrode delivers only weak amplitudes of electrocortical signals which remain sensitive to electronic noise and artifacts (10). In fact, in the last century, a common goal of the scientific community in the motor control field focused only on experiments regarding the relationships between human locomotion and cognition (11). The movement-related artifacts/noise contaminating the EEG recordings in motion-related experiments possibly explains the lack of investigations in actively walking participants. However, recent technological developments give rise to the expectation that EEG- and movement artifacts-related problems during human locomotion might be overcome. We will, therefore, briefly discuss some of these recent developments. We refer to a comprehensive overview of the theoretical and practical EEG and EEG-related principles (12, 13), since this topic is out of the scope of this work.

The interest in the supraspinal behavior during human locomotion has grown in the previous few decades and several studies started using functional near infrared spectroscopy (fNIRS) for the assessment of functional brain activation during human bipedalism (14, 15). fNIRS records the concentration levels of oxygenated hemoglobin (O_2_Hb) and has the advantage of being less prone to movement artifacts contamination. However, using fNIRS in this context has some limitations as well. To name a few, fNIRS is not able to provide information about brain structure for anatomical reference, the separation of the hemodynamic changes originating either from cerebral tissue or extra-cerebral tissues/structures is difficult (16), and finally most fNIRS devices are composed of a few channels only (i.e., optodes). A multimodal approach where fNIRS and EEG are used in combination can be possible as well. Concurrent EEG-fNIRS measurements can be collected with relative ease (17) and allow simultaneous investigation of the brain at multiple spatial and temporal scales (16).

Electroencephalography allows the direct measurement of post-synaptic neuronal activity, with very high time resolution but low spatial resolution. However, in recent years several improvements to overcome the spatial resolution limitation of EEG have been made. Enhanced EEG source reconstruction techniques (18–23) in combination with advanced statistical methods such as clustering of independent components (24–27) (see next paragraph for more details about this topic) and measure projection analysis (28) are an example. These developments have enhanced the achievable spatial resolution quality of EEG (28). Furthermore, EEG devices are available in several settings and with a number of channels up to 256 electrodes, which allows coverage of the entire human scalp.

The application of statistical methods to segregate cerebral and non-cerebral sources (e.g., noise, muscle activity, and eye blinks/movements) from the EEG recordings (29) through blind source separation (BSS) constitutes a major improvement for the assessment of brain behavior during human movement in ecologically valid scenarios. This BSS method, known most commonly as independent component analysis (ICA) (30, 31), has demonstrated its capability to separate overlapping and linearly mixed sources (both cerebral and non-cerebral) from the EEG recordings into mutually independent sources. That is, EEG recordings can be cleaned from non-cerebral components while preserving the cerebral ones (32, 33). This “revolutionary” method to clean EEG recordings from contamination of movement artifacts has more recently evolved in more advanced methods such as adaptive mixture-independent component analysis (34).

## Mobile Brain/Body Imaging: A New Promising Research Field

This aforementioned development has kicked-off a new research field, tagged as mobile brain/body imaging (MoBI) (35, 36), that translationally includes both knowledge from neuroscience and human movement sciences. MoBI introduces novel important opportunities allowing the investigation of the role of the CNS during human bipedal motion in natural environments (37, 38). Indeed, investigations using EEG approaches for studying human locomotion behavior has more recently shown an exponential increase, thus, unveiling several key aspects of cortical driven mechanisms of motor control in several human locomotion behavioral tasks, spanning from walking (39–46) to running (47) and from dancing (48) to cycling (49, 50). Even complex spatial navigation tasks (51) and virtual reality-based gait tasks (52) have been investigated with the help of this technique. Newer and deeper insights from more conventional types of analysis have been proposed, such as studies involving cognitive tasks (i.e., dual-task walking) in human bipedal motion (53–55).

A MoBI set-up allows an undefined number of body sensors to record human movement behavior concurrently with brain activity (e.g., by means of EEG). That is, the essence of the MoBI approach would require not only a mobile brain recording device but also simultaneous (and thus precisely triggered in time) several other measurement devices, in order to explore key aspects of the human locomotion linked to brain behavior. An example of such mobile wearable body sensors could be wireless surface electromyography (sEMG), 3D motion capture, inertial measurement units (IMUs), foot plantar pressure measurement systems, and/or eye tracker devices. A more comprehensive reading on wearable sensors options for MoBI studies can be found elsewhere (56).

## Brain/Muscle Connectivity in Human Locomotion: Standpoints, Limitations, and Future Directions

An important step in combining EEG with wearable body sensors has been performed by using sEMG. A recent MoBI study firstly showed that the motor cortex is actively committed in driving human locomotion such as walking (62). This was followed by several other similar studies (63–66). Together these studies represented the first direct and confirmatory evidence of supraspinal active controlling mechanisms, showing that brain activity is not solely constrained to a triggering role in human bipedal locomotion, but rather should be considered as being active during the whole motor execution phase, similar to the CPGs (67). This topic of investigation has been based on the assumption that the primary sensorimotor cortex (as assessed by means of EEG) is highly coupled (i.e., coherent) with muscle activity (as assessed by means of sEMG). That is, a measure of oscillatory synchronization between the primary sensorimotor cortex and spinal motor neurons activity (indirectly measured by sEMG), representing an actual measure of corticospinal interactions (68, 69). Coupled EEG–EMG analysis is often referred to as “corticomuscular coherence” (CMC) and has been first introduced by Baker in 1997 (70). CMC significant (71) values span from 0 to 1, where a higher number means higher coupling between EEG and EMG and, thus, a more efficient corticomuscular synchronization. Further details regarding this brain/muscle connectivity measure can be found elsewhere (72–74).

The promising potential of using wearable sensors in combination with brain imaging devices, investigating the communication between supraspinal and subcortical sites with concurrent kinematic events, is shown by recent studies involving peripherally attached (i.e., to the limbs) IMUs coupled with magnetoencephalography. These studies have shown promising results in the sense that using such combined techniques (i.e., corticokinematic coherence, CKC) is capable to parallel CMC in assessing the neuronal communication between brain and muscle(s) (57–61). CKC is a good example of how wearable sensors in combination with brain imaging devices might be used for the assessment of meaningful human bipedalism behavior. CKC, currently not yet employed within a MoBI framework, might represent an important opportunity for future MoBI studies aiming to explore the brain–muscle communication during human locomotion. Indeed, IMUs are rather simple and quick to use as well as less prone to be contaminated by motion-related artifacts, compared to sEMG.

A comparable measure of CMC, referred to as “intramuscular coherence” or “EMG-EMG coherence”, takes into consideration the common synchronized oscillatory drive to a pair of sEMG placed over the same muscle (75). Intramuscular coherence is supposed to reflect the neural drive from the primary motor cortex to the muscles (75). EMG–EMG coherence has been found to be quite reliable during treadmill walking, however, although the derived coherence variables can be considered to be reliable measures, large changes are needed to indicate a real difference in an individual level (76).

It can be reasoned that CMC reliability as measured during human locomotion tasks (i.e., walking) is also promising; however, thorough investigations of the psychometric properties of the measurement approach are needed to substantiate or refute this assumption. We are currently far from having precise and accurate measurements of neuronal communication during human bipedal motion and available results from studies employing CMC (or EMG–EMG) techniques should be interpreted with prudence. The reproducibility and reliability of CMC measurements during human bipedal motion is an important issue not yet clarified on both group and individual levels and should be topic of future studies.

Another issue that needs to be resolved relates to the consistently reported efferent cortical role during the gait cycle (62, 65, 66). So far only one study evaluated the CMC during overground walking, reporting that in this more “ecological” scenario also the afferent pathway is involved in and throughout human locomotion (66). This is a standpoint of importance, given that in many clinical conditions where gait disturbances are present (e.g., muscle weakness, Parkinson’s disease), the entire feedback–feedforward motor control loop seems to be impaired. However, CMC should be regarded as a surrogate measure of the actual and direct neuronal communication taking place in humans. For example, it may well be that CMC cannot be found in a (healthy) subject, because of limitations either in applied study protocols, in the methods of analysis employed or, more generally, because of subject-to-subject variability. Obviously, this does not mean absence of communication between brain and muscles; rather this is simply believed to be due to the fact that CMC applied during human bipedal motion assessment is still in its fledgling state of development and has several limitations that should be overcome.

Finally, future studies on CMC should consider to both establishing the reproducibility and reliability of CMC measurements as well as disentangling the causality in the connectivity measures (65, 66), possibly employing common CMC signal processing methods of analysis, thus enabling more consistent comparisons and to determine the most meaningful approach.

## Brain/Muscle Connectivity During Gait as Potential Novel “Biomarker” in Clinical Settings

It has been shown that CMC has the potential to distinguish the presence of neuromuscular disorders such as in upper motor neuron disease (77). However, an important feature that future investigations should take into account is represented by using CMC during gait as a potential biomarker of clinical diseases where human locomotion is impaired. An important opportunity might be represented by the age-related progressive decline in muscle mass/strength, namely *sarcopenia* (78). Sarcopenia has a prevalence of about 10–20% in community-dwelling older adults above 65 years and goes up to 30–50% in those aged above 80 years (79–81). Sarcopenia, recently recognized as a geriatric syndrome by the Centers for Disease Control and Prevention (USA), requires better diagnostic methods for its determination (81). The potential relevance of using CMC while walking for detecting sarcopenia is driven by the fact that recent studies demonstrated that muscle atrophy is rather a relatively small contributor to the loss of muscle strength (82). Mounting evidence points to changes in neurologic function and/or the intrinsic force-generating properties of skeletal muscle as contributors to muscle weakness and motor dysfunction (83). Indeed, CMC has been defined as *the hallmark of aging* (84), able to distinguish younger from older adults based on fine motor (or motor-cognitive) tasks (85). Combining CMC measures with active walking within a MoBI framework can be assumed to provide meaningful information about the locomotor system in older adults (86).

## Conclusion

In conclusion, the last two decades have opened a new and fascinating “door” on the motor control research field investigating neuronal communication in human locomotion. This field of research is still in its fledgling state, however, it is promising for revealing some aspects of the brain’s role in human locomotion. To increase the chances for replication both on group and individual human levels, studies using MoBI approaches in humans should specify parameters of the tests used, e.g., the exact procedures and instrumentation used, the duration of testing, and applied algorithms used for data analysis (87). An important feature is investigating CMC within the MoBI framework as a potential “biomarker” in neuromuscular disorders (or syndromes), where the need of finding novel and better diagnostics is warranted.

## Author Contributions

FG has developed the conception and design of the manuscript under the lead of EB. FG drafted a first version of the manuscript. EB substantially revised the manuscript. Both FG and EB have read, revised, and approved the final version of the manuscript.

## Conflict of Interest Statement

The authors declare that the research has been conducted in the absence of any commercial or financial relationships that could be construed as a potential conflict of interest.
